# Nanoscale structure of amyloid-β plaques in Alzheimer’s disease

**DOI:** 10.1038/s41598-019-41443-3

**Published:** 2019-03-26

**Authors:** Marta Querol-Vilaseca, Martí Colom-Cadena, Jordi Pegueroles, Raúl Nuñez-Llaves, Joan Luque-Cabecerans, Laia Muñoz-Llahuna, Jordi Andilla, Olivia Belbin, Tara L. Spires-Jones, Ellen Gelpi, Jordi Clarimon, Pablo Loza-Alvarez, Juan Fortea, Alberto Lleó

**Affiliations:** 1 Memory Unit, Department of Neurology, Institut d’Investigacions Biomèdiques Sant Pau - Hospital de Sant Pau, Universitat Autònoma de Barcelona, Barcelona, Spain; 2 Centro de Investigación Biomédica en Red en Enfermedades Neurodegenerativas (CIBERNED), Madrid, Spain; 3 ICFO-Institut de Ciències Fotòniques, The Barcelona Institute of Science and Technology, Castelldefels, Barcelona, Spain; 4 Neurological Tissue Bank of the Biobanc-Hospital Clinic-IDIBAPS, Barcelona, Spain; 5 The University of Edinburgh, UK Dementia Research Institute, Centre for Discovery Brain Sciences, Edinburgh, EH8 9JZ UK; 6 Institute of Neurology, Medical University of Vienna, Vienna, Austria

## Abstract

Soluble amyloid-β (Aβ) is considered to be a critical component in the pathogenesis of Alzheimer’s disease (AD). Evidence suggests that these non-fibrillar Aβ assemblies are implicated in synaptic dysfunction, neurodegeneration and cell death. However, characterization of these species comes mainly from studies in cellular or animal models, and there is little data in intact human samples due to the lack of adequate optical microscopic resolution to study these small structures. Here, to achieve super-resolution in all three dimensions, we applied Array Tomography (AT) and Stimulated Emission Depletion microscopy (STED), to characterize in postmortem human brain tissue non-fibrillar Aβ structures in amyloid plaques of cases with autosomal dominant and sporadic AD. Ultrathin sections scanned with super-resolution STED microscopy allowed the detection of small Aβ structures of the order of 100 nm. We reconstructed a whole human amyloid plaque and established that plaques are formed by a dense core of higher order Aβ species (~0.022 µm^3^) and a peripheral halo of smaller Aβ structures (~0.003 µm^3^). This work highlights the potential of AT-STED for human neuropathological studies.

## Introduction

Amyloid-β (Aβ) aggregation is believed to be a key initial pathophysiological event in Alzheimer’s disease (AD). Several *in vitro* and *in vivo* studies have shown that, under certain conditions, Aβ peptides are able to form small soluble oligomers that grow into protofibrils and finally into dense insoluble structures that accumulate in the brain in form of extracellular β-amyloid plaques^1–3^. There is growing evidence that soluble Aβ species are more toxic than fibrillar Aβ in causing neuronal loss and synaptic dysfunction^4–7^. Aβ oligomers can induce neuronal and synaptic damage through different mechanisms, such as inhibition of hippocampal long-term potentiation^8^, inhibition of exocytosis by impairing SNARE complex formation^9^, deregulation of NMDA-mediated calcium influx triggering synaptic collapse^10^ or the formation of membrane pores causing permeabilization and inducing neuronal death^11,12^.

The majority of cases of AD are sporadic (SAD) but in ~1% of cases the disease segregates with an autosomal dominant pattern (ADAD) and an early age of onset^13,14^. ADAD is caused by mutations in the amyloid precursor protein (APP) or in the two presenilin (*PSEN1* and *PSEN2*) genes^15,16^. Different studies have demonstrated that these mutations cause a chronic increase in the relative or absolute production of the 42-aa form of Aβ peptide (Aβ_42_) leading to the formation of oligomeric Aβ, fibrillar Aβ deposition and neurodegeneration^15,17^. In contrast, the mechanisms underlying Aβ accumulation in SAD patients are far more complex. The main hypothesis to explain Aβ deposition in SAD is the existence of a chronic imbalance between Aβ production and clearance as a result of aging and other risk factors^17–22^.

Although it is often assumed that the soluble Aβ species are the most toxic, the main properties such as size, morphology, structure and stability in human brain remain under study^23–26^. The majority of available data is based on experiments that used synthetic Aβ_42_ oligomers on cell cultures or animal models that overexpress mutant APP^5,6,27–30^ and few studies have investigated the role of non-fibrillar Aβ in intact human brain samples^31–39^.

The study of small structures such as non-fibrillar Aβ species in human brain samples has been challenging due to the limited resolution of immunohistochemistry using conventional microscopy. The lowest resolution obtained with optical microscopy is 200 nm, thus precluding any detailed characterization of those species. A recent study showed that the combination of new technologies such as focused ion beam milling and scanning electron microscopy (FIB/SEM) plus computational tools can be applied for the study of human amyloid plaques or synapses in the AD brain^40^. The use of hyperspectral Raman imaging has been also applied for the study of the biochemical components and β-sheet content of amyloid plaques and their surroundings^41^. In light microscopy, other techniques are also emerging for the study of small structures in brain tissue. One example is array tomography (AT), a technique that combines ultrathin sectioning of the tissue to increase the axial resolution with immunofluorescence, allowing quantitative analysis of high-resolution and large-field volumetric imaging of different specimens, such as synapses or pathological aggregates^42^. AT has demonstrated the ability to resolve Aβ synaptic deposits in AD brains^6^. Additionally, in the super-resolution (SR) fluorescence microscopy domain, Stimulated Emission Depletion (STED) microscopy is a technique that combines a high resolution confocal microscope with a high power donut-shaped depletion laser. The STED configuration uses the excitation and depletion lasers simultaneously increasing the lateral resolution by reducing the emission area selectively depleting the fluorescent molecules under the donut-shaped beam^43,44^.

In the present work, we propose a new tool to study the nanometric neuropathology of neurodegenerative diseases by combining ultrathin sections used in AT scanned with super-resolution STED microscopy. Applying AT-STED, we investigate the nanoscale architecture of non-fibrillar Aβ structures in human amyloid plaques. We also investigated the load and size of these non-fibrillar Aβ entities in post-mortem human brain tissue in ADAD (*PSEN1* mutation carriers) and early-onset sporadic AD (eoSAD) patients.

## Results

### Conventional immunohistochemistry reveals no differences between ADAD and eoSAD cases in total or non-fibrillar Aβ

We included a group of 14 patients with eoSAD, 10 patients with ADAD carrying a *PSEN1* mutation and 9 healthy controls. Demographic, clinical and genetic data are shown in Supplementary Table S1. Detailed neuropathological data of the cases is shown in Supplementary Table S2. As expected, eoSAD cases had a later age of onset and death and higher frequency of the *APOE*ε4 allele compared to ADAD cases.

**Figure 1:**
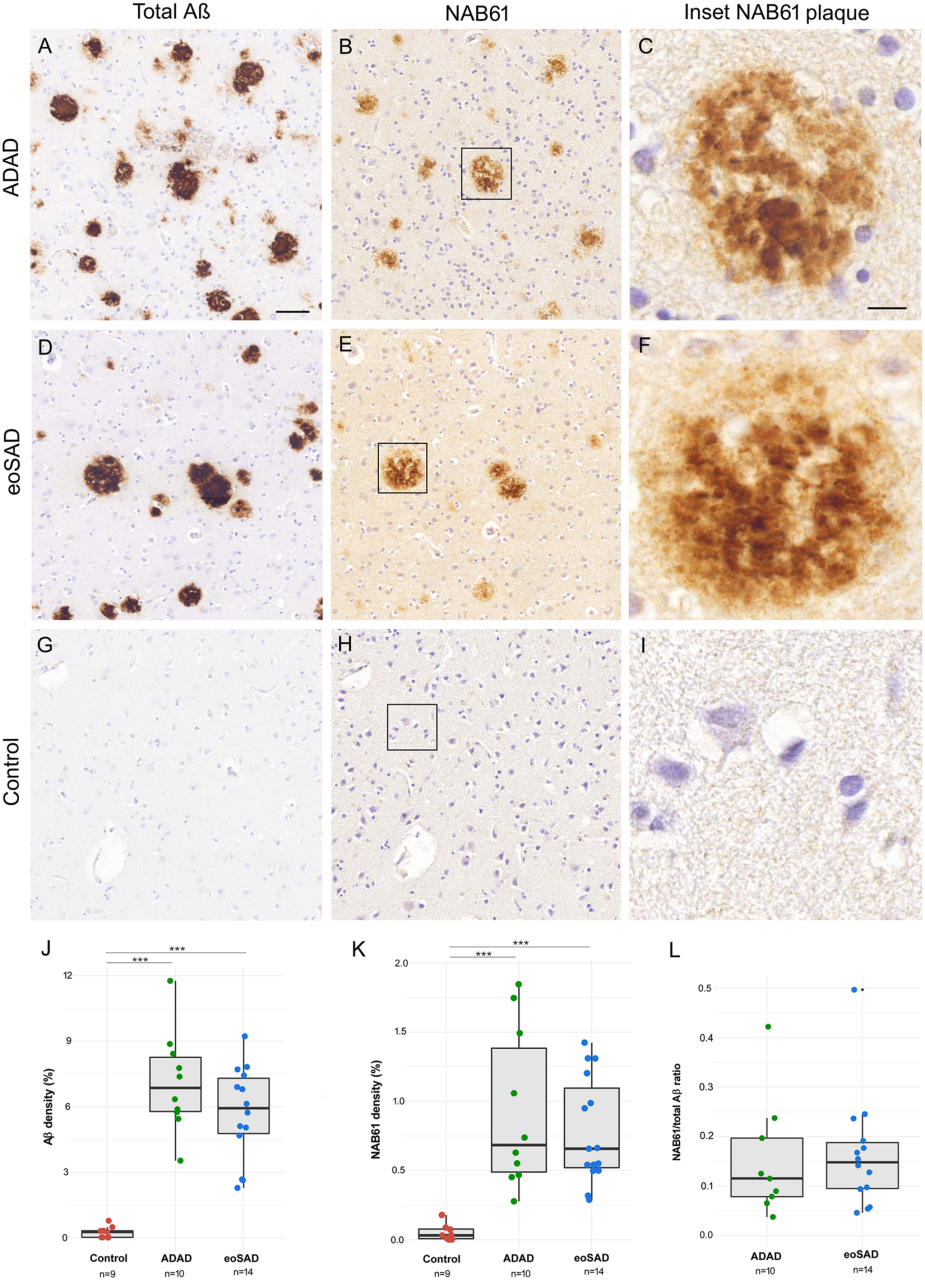
Quantification of total and non-fibrillar Aβ in ADAD and eoSAD cases. (**A**–**I**) Representative images of total Aβ and NAB61 immunoreactivity of ADAD, eoSAD and control cases. Strong immunoreactivity for both markers in consecutive sections from ADAD and eoSAD cases was found. Scale bar = 50 µm and 10 µm for inset. (**J**,**K**) Total Aβ and NAB61 densities were increased in both AD groups compared with controls. (**L**) No differences were found in the ratio NAB61/total Aβ between ADAD and eoSAD. ***p < 0.001.

We employed conventional immunohistochemistry of consecutive sections to examine the differences in total and non-fibrillar Aβ immunoreactivity (using the NAB61 antibody) between ADAD, eoSAD and control cases. We found higher amyloid load in ADAD and eoSAD cases (p < 0.001) compared to controls but without differences between the AD groups (Fig. 1).

### Improving spatial and axial resolution with AT and STED

**Figure 2:**
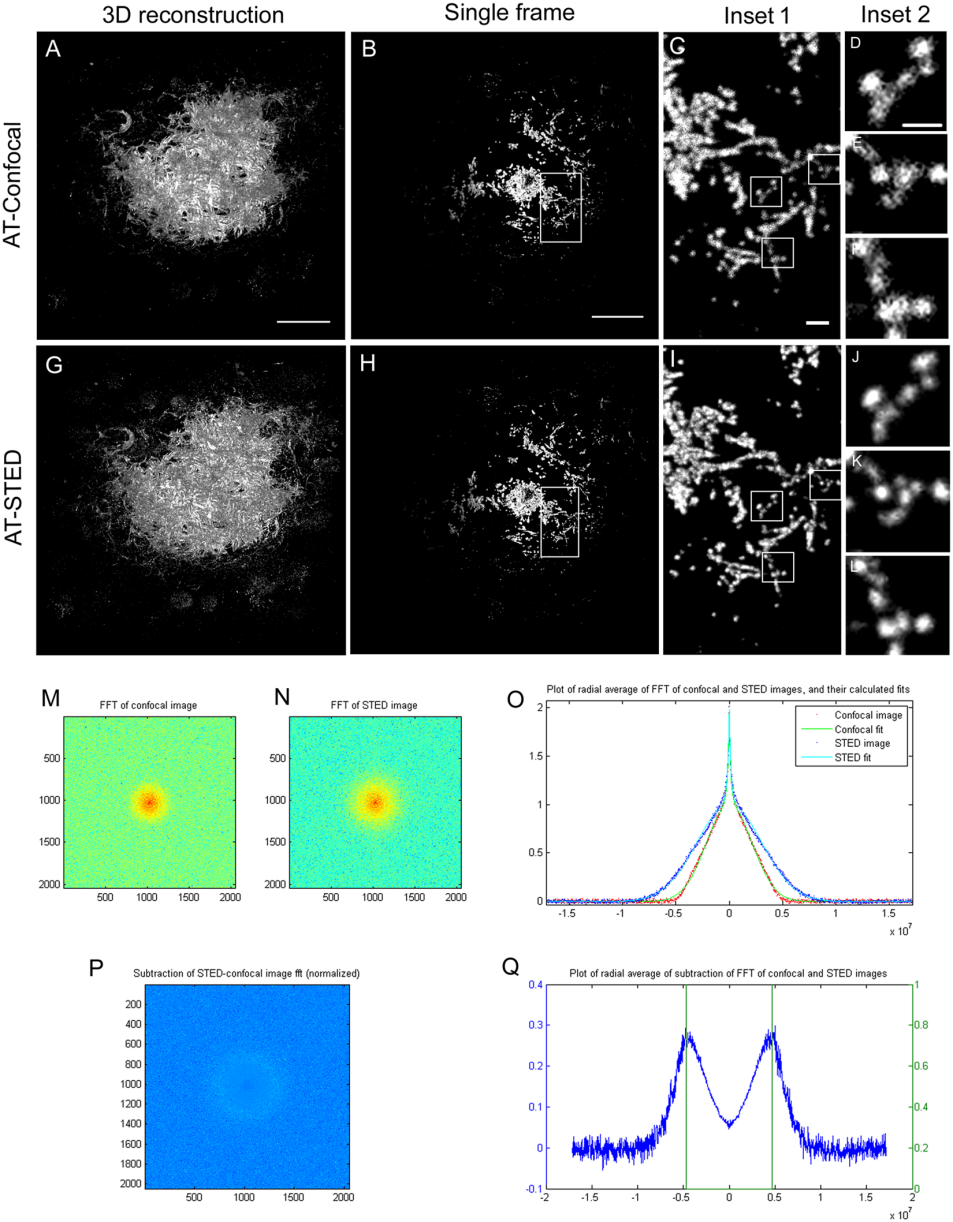
Comparison of the resolution between AT-Confocal and AT-STED modalities. (**A**,**G**) Maximal projection of a 3D reconstruction of an amyloid beta plaque in AT-Confocal and AT-STED respectively. (**B**,**H**) Image of a single slice of the amyloid-β plaque. (**C**–**F**; **I**–**L**) insets. (**M**,**N**) Scale bar = 10 µm, 1 µm for inset 1 and 0,5 µm for inset 2. Frequency domain representation of B and H. (**O**) Radial average of the frequency information for the AT-Confocal and AT-STED cases. (**P**,**Q**) Difference between of the radial frequency information between modalities. Green lines show the theoretical diffraction limit for 1.4NA objective.

We next investigated the potential of combining two microscopy techniques to investigate non-fibrillar Aβ structures in human AD brains. AT provides improved resolution in the axial plane by obtaining 70 nm thick tissue sections. Due to the special fixation and tissue processing requirements for this method, the availability of samples for this type of studies is limited. In order to obtain nanometric resolution in the lateral directions here we combined AT with STED. STED microscopy combines a high resolution confocal microscope with a high power depletion laser. This laser has been engineered to obtain a donut-shaped focal-spot and it is scanned simultaneously with the excitation laser. This configuration enables stimulated depletion emission on the molecules under the donut-shaped beam. Thus, the emitted light in this outer region of the excitation beam is forced to emit at the wavelength of the STED laser. Using adequate spectral filtering, the light generated in the center of the donut is then collected for analysis which is determined by the power of the STED laser. We acquired images of amyloid-β plaques with an STED microscope using both confocal and STED configurations. The combination of AT and the settings of STED used in this experiment yielded a minimum effective voxel size of 70 × 100 × 100 nm. This voxel size allowed us to resolve smaller non-fibrillar Aβ structures in comparison with using AT alone (Fig. 2). A 3D reconstruction of a full amyloid β plaque of ~50 µm of diameter is shown in Fig. 2. The use of AT-STED microscopy provided increased resolution of the amyloid-β aggregates compared with high resolution confocal microscopy. A tool published by Merino *et al*.^44^, enables to obtain an objective parameter, in which the sample is also considered, that can be used to determine the optimal settings for the STED image acquisition. Using this tool, we demonstrated an extension in the frequencies that allowed identifying objects down to 110 nm; in contrast, with AT alone the size of the smallest structure detected in the lateral plane was 220 nm.

### The combination of AT and STED allows the identification of smaller non-fibrillar Aβ structures

**Figure 3:**
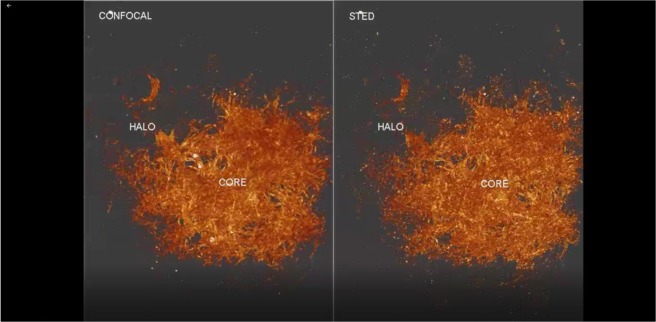
Nanoscale architecture of an entire human amyloid plaque using AT-STED. Reconstruction of 368 consecutive sections of an amyloid plaque from a SAD case. Zoom during the video shows the non-fibrilar Aβ structures better resolved in STED compared to confocal microscopy.

We applied AT-STED to 368 consecutive sections to obtain a detailed architecture of a whole human amyloid β plaque at a nanoscale resolution (Fig. 3).

**Figure 4:**
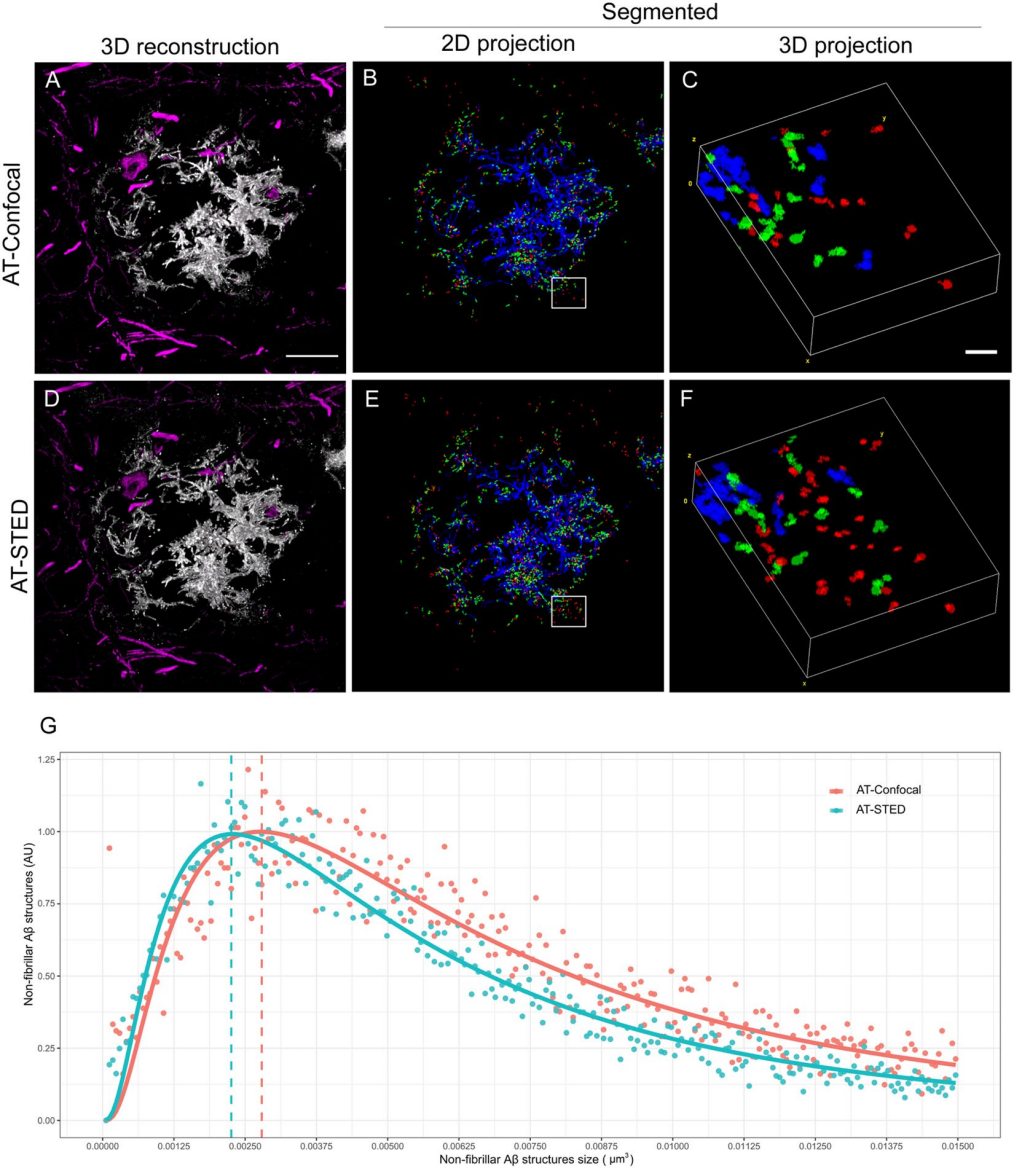
Distribution and quantification of non-fibrillar Aβ structures using AT-Confocal vs AT-STED. (**A**,**D**) Representative images of a human amyloid beta plaque and the surrounding neuronal structures (neurofilament light magenta) using (**A**) AT alone or (**D**) AT-STED. (**B**,**E**) Images of a segmented 2D projection of the Aβ plaque and (**C**,**F**) the respective 3D insets. Each color indicates a different Aβ structure’s size: large (>0,015 µm^3^; blue), medium (>0,006 < 0,015 µm^3^; green) and small (<0,006 µm^3^; red). Detection of a dense core formed by large Aβ structures and a halo around the plaque formed by medium and small Aβ entities. Scale bar = 10 µm and 1 µm for the inset. (**G**) AT-Confocal and AT-STED distribution of NAB61 structures.

We next selected randomly amyloid β plaques (n = 13) from two AD cases (one ADAD and one SAD) and captured images using both confocal and STED configurations to examine the size and distribution of NAB61-immunoreactive Aβ structures (a total of 18.000 objects). We also immunostained for neurofilaments to visualize the neuronal structures around the plaque (Fig. 4A,D). We divided the objects into three categories: small (<0,006 µm^3^; red), medium (>0,006 <0,015 µm^3^; green) and large (>0,015 µm^3^; blue). We obtained a distinguished dense core formed by higher order Aβ structures (blue) and a halo of medium and small non-fibrillar Aβ entities (green and red) surrounding the plaque (Fig. 4). Using the combination of AT-STED compared to AT alone, we observed an increase of all Aβ structures (Fig. 4). Quantitative analyses of the distributions confirmed that the combination of AT-STED allows detecting smaller Aβ structures compared with AT alone (Fig. 4).

### Higher levels of non-fibrillar Aβ structures in ADAD than in SAD

**Figure 5:**
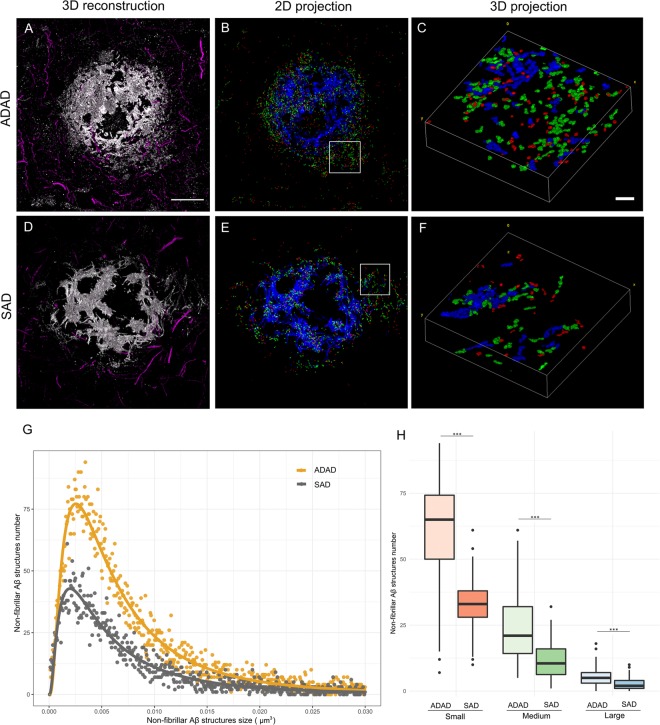
Non-fibrillar Aβ structures are increased in an ADAD case. (**A**–**F**) Representative images of non-fibrillar Aβ structures distribution and size identified in plaques of an ADAD (n = 8) and a SAD (n = 5) case using AT-STED. Neurofilament light protein (magenta) was stained to visualize the neuronal distribution around the plaques. Scale bar = 10 µm and 1 µm for the inset. (**G**) ADAD and SAD distribution of NAB61 structures number by size. Differences were found between both distributions (p < 0.001). (**H**) All Aβ entities were significantly increased in ADAD compared to SAD case (p < 0.001).

Taking advantage of the enhanced resolution, we applied AT-STED to investigate potential differences in non-fibrillar Aβ structures between an ADAD and a SAD case. We stratified the objects depending on the presence of *PSEN1* mutation and analyzed the distribution, size, proportion and quantity of non-fibrillar Aβ structures (total of ~18.000 objects). We observed an increase in the number of Aβ structures for all sizes (small, medium and large) in the ADAD case compared with the SAD case (p < 0.001) (Fig. 5).

## Discussion

This study combines two microscopy techniques to examine non-fibrillar Aβ structures in human amyloid plaques in the brain. Using this enhanced resolution we demonstrate that the distribution of Aβ assemblies consists of a dense core of higher order Aβ species surrounded by a peripheral halo of small Aβ structures. The combination of AT and STED allowed detecting non-fibrillar Aβ forms of at least 100 nm. Finally, we provide preliminary evidence of higher levels of Aβ structures in an ADAD case compared to a SAD case.

Several studies have focused on the characterization of Aβ oligomers in AD^6,45–47^. Electrophysiological and biochemical experiments have suggested that soluble Aβ oligomers correlate with disease severity^48,49^ and that there is an inverse correlation between the size of Aβ assemblies and their toxicity in multiple *in vitro* and *in vivo* models^3,6,7^. The potential structure, size, conformation, aggregation and induction of neurotoxicity of Aβ oligomers in AD pathogenesis has been thoroughly investigated through the application of several advanced technologies such as ion mobility-based mass spectrometry^25^, atomic force microscopy^50,51^, solid-state nuclear magnetic resonance^52^ and X-ray microdiffraction^53^. Other studies have used FIB/SEM to investigate the relationship between amyloid plaques and the synaptic organization in human AD brains^40^.

Here, we combined AT and STED to improve the resolution obtained with conventional confocal microscopy (x, y: 250 nm and z: 700 nm) and the detection of non-fibrillar Aβ structures in human amyloid plaques. AT achieves a resolution of 70 nm on the axial plane, while STED microscopy can increase the lateral resolution. In our case the addition of STED increased the lateral resolution up to 100 nm. This technique has been applied to investigate in detail the cellular mechanisms underlying dendritic spine plasticity^54,55^, glial processes^56^ or α-synuclein synaptic aggregation^57^ among others. Using the enhanced resolution of AT-STED we were able to reconstruct a whole human amyloid plaque and examined in detail the nanoscale distribution and size of the non-fibrillar Aβ species. AT-STED allows detection of Aβ structures of at least 100 nm, an undetectable size range for immunohistochemical assays due to the resolution limit of light and confocal microscopes (~250 nm)^43,58^. The study revealed that human amyloid plaques consist of a dense core of NAB61 large immunoreactive structures (~0.022 µm^3^) and a peripheral halo of medium and small non-fibrillar Aβ entities (~0.01 µm^3^ and ~0.003 µm^3^ respectively). These results are in agreement with a study that used the AT technique to study NAB61 immunoreactivity in an animal model of AD^5^. However, we were able to detect non-fibrillar Aβ structures 2 times smaller using AT-STED in human tissue.

In addition, we show preliminary evidence that non-fibrillar Aβ structures were increased in an ADAD case compared to a SAD case in human brain tissue. It is known that oligomeric Aβ accumulation induces neuronal cell loss^27^, astrocytic dysfunction^59^ and synaptic toxicity^6,33^, and it is possible that higher levels of non-fibrillar Aβ are a characteristic of ADAD and may be the reflection of abnormal APP processing^17^. Interestingly, this result was not detectable in conventional immunoassays pointing out the importance of implementing super-resolution techniques for detailed neuropathological studies.

The strengths of this work are the inclusion of two microscopy techniques for the study of human brain samples; and the implementation of computational tools for image processing and analysis. The main limitations are the small sample size for AT-STED due to fact that the requirement of fresh tissue makes these samples extremely scarce. Second, we only used one antibody to detect non-fibrillar Aβ. However, in our hands specific antibodies for oligomeric Aβ suitable for human tissue are exceedingly rare and NAB61 has been extensively characterized^5,33,60^.

In conclusion, as a proof of concept our study shows that the combination of AT and STED can be successfully applied to investigate non-fibrillar Aβ structures in AD human brain. The obtained nanoscale architecture of human amyloid plaques reveals a dense core with a peripheral halo and we provide evidence of higher levels of non-fibrillar Aβ species in ADAD compared to SAD. Additional studies are needed to further investigate the potential relevance of these assemblies in the pathogenesis of the disease. This new tool proposed opens an important door for the neuropathology field allowing the characterization of aggregates or structures at a nanometric scale as potential therapeutic targets.

## Materials and Methods

### Standard protocol approval and patient consent

We obtained written informed consent from all brain donors and/or next of kin for the use of brain tissue for research. The study was approved by the local ethics committee of Hospital de Sant Pau, Barcelona, Spain. All research was performed in accordance with relevant guidelines and regulations.

### Postmortem human brain samples

Brain samples for immunohistochemical assays were provided by the Neurological Tissue Bank (NTB) of the Biobanc-Hospital Clínic-IDIBAPS and processed as previously described^61^ and as internationally recommended^62^. The study group consisted of 34 subjects: 10 patients with *PSEN1* mutations, 14 eoSAD patients with an age of onset <65 years and a group of 9 healthy controls. Fresh brain tissue from one ADAD (*PSEN1* G206D, age 63) and one SAD case (age 90) was processed for AT as previously described^57,63^ and included in our collection.

### Immunohistochemistry, image acquisition and analysis

Immunohistochemistry was performed on 5-μm-thick consecutive sections of occipital cortex on an automated stainer (DAKO Autostainer Plus; DAKO, Glostrup, Denmark) using the following primary antibodies: mouse monoclonal anti-amyloid beta clone 6F/3D (dilution 1:400, DAKO, Glostrup, Denmark) and mouse anti-NAB61 (dilution 1:250, a kind gift from Virginia Lee, University of Pennsylvania, Philadelphia, USA). Reaction was visualized by the EnVision + system peroxidase procedure (DAKO, Glostrup, Denmark). Full-section scans were obtained with Pannoramic MIDI II (3DHistech, Budapest, Hungary) using a 40x objective. Cortical grey matter of each case was manually delimitated with pannoramic viewer software (3DHistech, Budapest, Hungary). An adaptation of the algorithm previously described^64^ was developed to quantify total Aβ and NAB61 immunoreactivity with MATLAB software (The Math Works, Inc., Natick, MA, USA). Specifically, a colour deconvolution was used to split the channels and a mean local filter was then applied to segment the structures (Supplementary Fig. S1).

### Array Tomography

Brain samples for AT were processed using previously described methods^57,63^. Briefly, 70 nm-thick sections were obtained using an ultramicrotome (Leica Microsystems) equipped with an Ultra Jumbo Diamond Knife 35° (Diatome) from LR white embedded tissue blocks. The ribbons were stained as previously described^63^ and incubated with Tris-Glycine solution 5′ at RT followed by a blocking of unspecific antigens with a cold water fish blocking buffer (Sigma-Aldrich) for 30 min. Sections were then incubated for 2 hours with the following primary antibodies: mouse anti-NAB61 (dilution 1:50, kindly provided by Virginia Lee, University of Pennsylvania, Philadelphia, USA) and rabbit anti-Neurofilament Light (dilution 1:50, #2837, Cell Signaling). After TBS washings, secondary fluorescent antibodies Alexa 488 and Alexa 555 (dilution 1:50, Invitrogen) were applied for 30’. Sections were washed with TBS and samples were stained with Hoechst 33258 (dilution 1:100, Life Technologies) for 5 min for nuclei visualization. Finally, coverslips were mounted on microscope slides with Immu-Mount (Fisher Scientific) mounting medium.

### STED and confocal image acquisition

The acquisition of confocal and STED images has been performed using a Leica STED CW microscope (Leica Microsystems). The excitation wavelength was 488 nm and the spectral detector window has been set from 495 nm to 588 nm. The objective used was a Leica HCX PL APO CS 100.0x STED with 1.4 NA. The 592 nm STED laser was set to 85% and the detectors used were Hybrid detectors in photon counting mode. The scanning speed was 1000 Hz per line and each acquisition has been obtained accumulating six times per line and averaging two acquisitions per image. Finally, these settings provided a set of images of 2048 × 2048 with effective pixel dwell time of 5.9 µs.

### Image processing and analysis

For the analysis of AT images, MATLAB software (The Math Works, Inc., Natick, MA, USA) was used. Image stacks of each channel comprising all 70 nm consecutive sections were first registered using a rigid registration followed by an affine registration of a reference channel. Aligned sections were segmented using an automated mean local thresholding. Additionally, objects that were not in at least two consecutive sections or that were less than 3px were considered background and removed. After identification of three-dimensional structures, the density and size of individual entities were quantified. In order to perform the 3D visualization of the human amyloid plaque, the 2D images were stacked in a whole 3D reconstruction.

### Statistical analysis

R software (version 3.2.5, www.r-project.org) was used to assess the statistical analysis. Due to the non-normal distribution of the data, non-parametric tests were applied. For immunohistochemical assays, Kruskal-Wallis with Dunn’s post testing were used to detect differences in NAB61 and total Aβ levels between groups and a Mann-Whitney test was used for the ratio. To assess the differences between the ADAD case and the SAD case, an unpaired Mann-Whitney test was used.

## Supplementary information

Figure 3

Supplementary material

## Data Availability

All data generated or analysed during this study are included in this published article (and its Supplementary Information files).

## References

[1] Walsh DM, Selkoe DJ (2007). A beta oligomers - a decade of discovery. J Neurochem..

[2] Kayed R, Lasagna-Reeves CA (2013). Molecular mechanisms of amyloid oligomers toxicity. J Alzheimers Dis..

[3] Viola KL, Klein WL (2015). Amyloid β oligomers in Alzheimer’s disease pathogenesis, treatment, and diagnosis. Acta Neuropathol..

[4] Lacor PN (2007). Abeta oligomer-induced aberrations in synapse composition, shape, and density provide a molecular basis for loss of connectivity in Alzheimer’s disease. J Neurosci..

[5] Koffie RM (2009). Oligomeric amyloid beta associates with postsynaptic densities and correlates with excitatory synapse loss near senile plaques. Proc Natl Acad Sci USA.

[6] Pickett EK (2016). Non-Fibrillar Oligomeric Amyloid-β within Synapses. J Alzheimers Dis..

[7] Sengupta U, Nilson AN, Kayed R (2016). The Role of Amyloid-β Oligomers in Toxicity, Propagation, and Immunotherapy. EBioMedicine..

[8] Walsh DM (2002). Naturally secreted oligomers of amyloid beta protein potently inhibit hippocampal long-term potentiation *in vivo*. Nature..

[9] Yang Y (2015). Amyloid-β Oligomers may mpair SNARE-mediated exocytosis by direct binding to syntaxin 1a. 2015. Cell Rep..

[10] Arbel-Ornath M (2017). Soluble oligomeric amyloid-β induces calcium dyshomeostasis that precedes synapse loss in the living mouse brain. Mol Neurodegener..

[11] Zhao LN, Long H, Mu Y, Chew LY (2012). The toxicity of amyloid β oligomers. Int J Mol Sci..

[12] Serra-Batiste M (2016). Aβ42 assembles into specific β-barrel pore-forming oligomers in membrane-mimicking environments. Proc Natl Acad Sci USA.

[13] Lleó A, Castellví M, Blesa R, Oliva R (2002). Uncommon polymorphism in the presenilin genes in human familial Alzheimer’s disease: not to be mistaken with a pathogenic mutation. Neurosci Lett..

[14] Lleó A, Berezovska O, Growdon JH, Hyman BT (2004). Clinical, pathological, and biochemical spectrum of Alzheimer disease associated with PS-1 mutations. Am J Geriatr Psychiatry..

[15] Bateman RJ (2011). Autosomal-dominant Alzheimer’s disease: a review and proposal for the prevention of Alzheimer’s disease. Alzheimers Res Ther..

[16] Lleó A (2002). Frequency of mutations in the presenilin and amyloid precursor protein genes in early-onset Alzheimer disease in Spain. Arch Neurol..

[17] Pera M (2013). Distinct patterns of APP processing in the CNS in autosomal-dominant and sporadic Alzheimer disease. Acta Neuropathol..

[18] Hebert SS (2008). Loss of microRNA cluster miR-29a/b-1 in sporadic Alzheimer’s disease correlates with increased BACE1/beta-secretase expression. Proc Natl Acad Sci USA.

[19] Li R (2004). Amyloid beta peptide load is correlated with increased beta-secretase activity in sporadic Alzheimer’s disease patients. Proc Natl Acad Sci USA.

[20] Yang LB (2003). Elevated beta-secretase expression and enzymatic activity detected in sporadic Alzheimer disease. Nat Med..

[21] Hata S (2011). Alternative processing of gamma-secretase substrates in common forms of mild cognitive impairment and Alzheimer’s disease: evidence for gammasecretase dysfunction. Ann Neurol.

[22] Mawuenyega KG (2010). Decreased clearance of CNS beta-amyloid in Alzheimer’s disease. Science..

[23] Yang T, Li S, Xu H, Walsh DM, Selkoe DJ (2017). Large Soluble Oligomers of Amyloid β-Protein from Alzheimer Brain Are Far Less Neuroactive than the smaller oligomers to which they dissociate. J Neurosci..

[24] Breydo L, Uversky VN (2015). Structural, morphological, and functional diversity of amyloid oligomers. FEBS Lett..

[25] Gessel MM, Bernstein S, Kemper M, Teplow DB, Bowers MT (2012). Familial Alzheimer’s disease mutations differentially alter amyloid β-protein oligomerization. ACS Chem Neurosci..

[26] Nag S (2011). Nature of the amyloid-beta monomer and the monomer-oligomer equilibrium. J Biol Chem..

[27] DaRocha-Souto B (2011). Brain oligomeric β-amyloid but not total amyloid plaque burden correlates with neuronal loss and astrocyte inflammatory response in amyloid precursor protein/tau transgenic mice. J Neuropathol Exp Neurol..

[28] DiChiara T (2017). Alzheimer’s Toxic Amyloid Beta Oligomers: Unwelcome Visitors to the Na/K ATPase alpha3 Docking Station. Yale J Biol Med..

[29] Shafrir Y, Durell SR, Guy HR (2010). Beta-barrel models of soluble amyloid beta oligomers and annular protofibrils. Proteins..

[30] Marsh J, Bagol SH, Williams RSB, Dickson G, Alifragis O (2017). Synapsin I phosphorylation is dysregulated by beta-amyloid oligomers and restored by valproic acid. Neurobiol Dis..

[31] Walsh DM, Tseng BP, Rydel RE, Podlisny MP, Selkoe DJ (2000). The oligomerization of amyloid beta-protein begins intracellularly in cells derived from human brain. Biochemistry..

[32] Gouras GK, Tampellini D, Takahashi RH, Capetillo-Zarate E (2010). Intraneuronal beta-amyloid accumulation and synapse pathology in Alzheimer’s disease. Acta Neuropathol..

[33] Koffie RM (2012). Apolipoprotein E4 effects in Alzheimer’s disease are mediated by synaptotoxic oligomeric amyloid-beta. Brain..

[34] Perez-Nievas BG (2013). Dissecting phenotypic traits linked to human resilience to Alzheimer’s pathology. Brain..

[35] Bodani RU (2015). Antibody against Small Aggregated Peptide Specifically Recognizes Toxic Aβ-42 Oligomers in Alzheimer’s Disease. ACS Chem. Neurosci..

[36] Bilousova T (2016). Synaptic Amyloid-β oligomers precede p-Tau and differentiate high pathology control cases. Am. J. Pathol..

[37] Kumar S (2016). Phosphorylation of the amyloid beta-peptide at ser26 stabilizes oligomeric assembly and increases neurotoxicity. Acta Neuropathol..

[38] Savioz A (2016). A Study of Abeta Oligomers in the Temporal Cortex and Cerebellum of Patients with Neuropathologically Confirmed Alzheimer’s Disease Compared to Aged Controls. Neurodegener-Dis..

[39] Li S (2018). Decoding the synaptic dysfunction of bioactive human AD brain soluble Aβ to inspire novel therapeutic avenues for Alzheimer’s disease. Acta Neuropathol Commun..

[40] Blazquez-Llorca L, Merchán-Pérez Á, Rodríguez JR, Gascón J, DeFelipe J (2013). FIB/SEM technology and Alzheimer’s disease: three-dimensional analysis of human cortical synapses. J Alzheimers Dis..

[41] Michael R (2017). Hyperspectral Raman imaging of neuritic plaques and neurofibrillary tangles in brain tissue from Alzheimer’s disease patients. Sci Rep..

[42] Micheva KD, Smith SJ (2007). Array tomography: a new tool for imaging the molecular architecture and ultrastructure of neural circuits. Neuron..

[43] Schermelleh L, Heintzmann R, Leonhardt H (2010). A guide to super-resolution fluorescence microscopy. J Cell Biol..

[44] Merino D (2017). STED imaging performance estimation by means of Fourier transform analysis. Biomed. Opt. Express..

[45] Kayed R (2003). Common structure of soluble amyloid oligomers implies common mechanism of pathogenesis. Science..

[46] Stroud JC, Liu C, Teng PK, Eisenberg D (2012). Toxic fibrillar oligomers of amyloid-β have cross-β structure. Proc. Natl. Acad. Sci. USA.

[47] Kotler SA (2015). High-resolution NMR characterization of low abundance oligomers of amyloid-β without purification. Sci Rep..

[48] McLean CA (1999). Soluble pool of Abeta amyloid as a determinant of severity of neurodegeneration in Alzheimer’s disease. Ann Neurol.

[49] Tomic JL, Pensalfini A, Head E, Glabe CG (2009). Soluble fibrillar oligomer levels are elevated in Alzheimer’s disease brain and correlate with cognitive dysfunction. Neurobiol Dis.

[50] Economou NJ (2016). Amyloid β-Protein Assembly and Alzheimer’s Disease: Dodecamers of Aβ42, but Not of Aβ40, Seed Fibril Formation. J Am Chem Soc..

[51] Ungureanu AA (2016). Amyloid beta oligomers induce neuronal elasticity changes in age-dependent manner: a force spectroscopy study on living hippocampal neurons. Sci Rep..

[52] Qiang W, Yau WM, Lu JX, Collinge J, Tycko R (2017). Structural variation in amyloid-β fibrils from Alzheimer’s disease clinical subtypes. Nature..

[53] Liu J (2016). Amyloid structure exhibits polymorphism on multiple length scales in human brain tissue. Sci Rep..

[54] Wegner W (2017). *In vivo* mouse and live cell STED microscopy of neuronal actin plasticity using far-red emitting fluorescent proteins. Sci Rep..

[55] Berning S, Willig KI, Steffens H, Dibaj P, Hell SW (2012). Nanoscopy in a living mouse brain. Science..

[56] Bethge P, Chéreau R, Avignone E, Marsicano G, Nägerl UV (2013). Two-photon excitation STED microscopy in two colors in acute brain slices. Biophys J..

[57] Colom-Cadena M (2017). Synaptic phosphorylated α-synuclein in dementia with Lewy bodies. Brain..

[58] Pawley, J.B. Handbook of Biological Confocal Microscopy. (Springer Science + Business Media, 2006).

[59] Diniz LP (2017). Astrocyte Transforming Growth Factor Beta 1 Protects Synapses against Aβ Oligomers in Alzheimer’s Disease Model. J Neurosci..

[60] Lee EB (2006). Targeting amyloid-beta peptide (Abeta) oligomers by passive immunization with a conformation-selective monoclonal antibody improves learning and memory in Abeta precursor protein (APP) transgenic mice. J Biol Chem..

[61] Colom-Cadena M (2013). Confluence of α-synuclein, tau, and β-amyloid pathologies in dementia with Lewy bodies. J Neuropathol Exp Neurol..

[62] Montine TJ (2012). National Institute on Aging-Alzheimer’s Association guidelines for the neuropathologic assessment of Alzheimer’s disease: a practical approach. Acta Neuropathol..

[63] Kay KR (2013). Studying synapses in human brain with array tomography and electron microscopy. Nat Protoc..

[64] Querol-Vilaseca M (2017). YKL-40 (Chitinase 3-like I) is expressed in a subset of astrocytes in Alzheimer’s disease and other tauopathies. J Neuroinflammation..

