# Supplementary material for: Nanoscale structure of amyloid-β plaques in Alzheimer’s disease
Source: Sci Rep. 2019 Mar 26;9:5181. doi: 10.1038/s41598-019-41443-3 (PMC6435662; doi:10.1038/s41598-019-41443-3)
Supplement: Supplementary file 2 — Supplementary material [file 41598_2019_41443_MOESM2_ESM.pdf]

Nanoscale structure of amyloid-β plaques in Alzheimer's disease

Marta Querol-Vilaseca[1,2]; Martí Colom-Cadena[1,2]; Jordi Pegueroles[1,2]; Raúl Nuñez-Llaves[1,2]; Joan Luque-Cabecerans[1,2]; Laia Muñoz-Llahuna[1,2]; Jordi Andilla[3]; Olivia Belbin[1,2]; Tara L Spires-Jones[5]; Ellen Gelpi[4,6]; Jordi Clarimon[1,2]; Pablo Loza-Alvarez[3]; Juan Fortea[1,2]; Alberto Lleó[1,2 *]

[1] Memory Unit, Department of Neurology, Institut d'Investigacions Biomèdiques Sant Pau - Hospital de Sant Pau, Universitat Autònoma de Barcelona, Barcelona, Spain.

[2] Centro de Investigación Biomédica en Red en Enfermedades Neurodegenerativas (CIBERNED), Madrid, Spain.

[3] ICFO-Institut de Ciències Fotòniques, The Barcelona Institute of Science and Technology, Castelldefels, Barcelona, Spain.

[4] Neurological Tissue Bank of the Biobanc-Hospital Clinic-IDIBAPS, Barcelona Spain.

[5] The University of Edinburgh, UK Dementia Research Institute, Centre for Discovery Brain Sciences, Edinburgh, EH8 9JZ, UK.

[6] Institute of Neurology, Medical University of Vienna, Vienna, Austria.

*** Corresponding author:** Alberto Lleó, MD, PhD. Neurology Department. Hospital de Sant Pau. Sant Antoni M. Claret 167, 08025 Barcelona, Spain. Phone: +34 93 5565986; Fax: +34 935565602; E-mail: alleo@santpau.cat; ORCID: 0000-0002-2568-5478

SUPPLEMENTAL MATERIAL

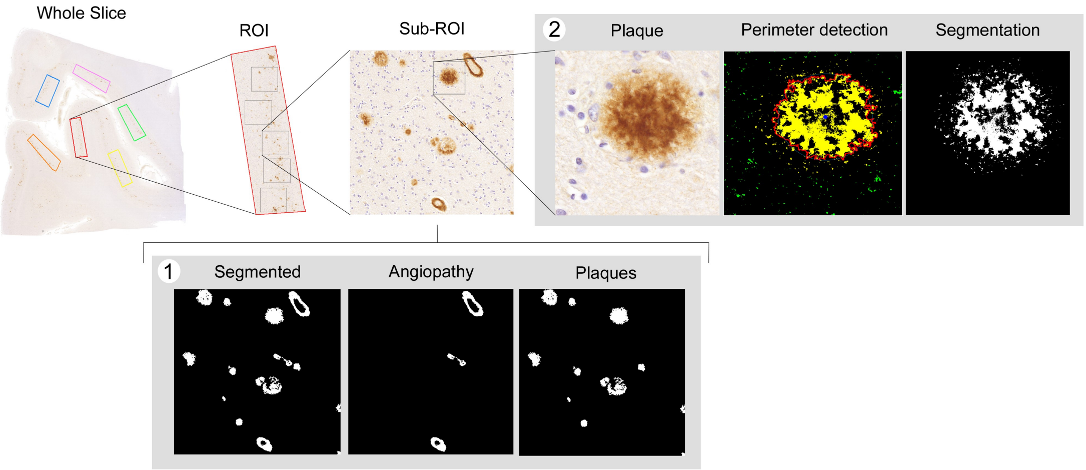

Supplementary Figure S1. Image processing pipeline. Whole slice scans are obtained and random regions of interest are manually delimitated. The application of an in-house algorithm allows two different analyses. 1) Quantification of total Aβ or NAB61 immunoreactivity excluding amyloid angiopathy. 2) Quantification of soluble NAB61+ structures around plaques.

Supplementary Table S1. Demographic, clinical and genetic data of eoSAD and ADAD patients.

	**ADAD (N =10)**	**eoSAD (N =14)**	**p value**
Gender = M (%)	7 (70.0)	6 (42.9)	0.368
Mean age at onset (mean (SD))	43.80 (6.16)	51.36 (3.52)	**0.001**
Mean age at death (mean (SD))	54.90 (5.69)	60.79 (4.10)	**0.007**
Mean disease duration (mean (SD))	11.10 (4.41)	9.43 (3.25)	0.296
APOE ε4 allele carriers = n (%)	2 (25.0)*	8 (66.7)*	0.171

*Calculated without missing data (ADAD n=8 and eoSAD n=12). M: male; SD: standard deviation.

Supplementary Table S2. Neuropathologic data of eoSAD and ADAD cases.

Case #	Mutation	Gender	Thal Aβ phase	Braak NF stage	CERAD	Age at onset	Age at death	APOE genotype	PMI (h)
PSEN1 mutation carriers									
1	V89L	M	5	V	freq	45	54	23	7,5
2	V89L	M	5	VI	freq	48	57	23	9.5
3	E120G	M	5	VI	freq	34	44	33	5.5
4	M139T	M	5	V	freq	47	64	33	14.7
5	M139T	M	5	VI	freq	48	57	33	15.2
6	M139T	M	5	VI	freq	45	53	33	5.3
7	M139T	F	5	VI	freq	38	48	23	5,25
8	P264L	F	5	VI	freq	45	56	44	6
9	P264L	M	5	VI	freq	53	60	34	7.2
10	G206D	M	5	VI	freq	37	44	34	19.2
Sporadic Early-onset AD									
11	-	M	4	V	freq	51	65	44	18
12	-	M	4	VI	freq	52	65	33	8
13	-	M	4	VI	freq	52	60	44	10,25
14	-	F	5	VI	freq	51	60	33	5
15	-	M	5	VI	freq	46	55	33	4,5
16	-	F	4	VI	freq	50	63	33	6,5
17	-	F	5	VI	freq	56	67	44	4
18	-	F	5	VI	freq	54	62	44	6
19	-	F	5	VI	freq	58	66	43	12,5
20	-	F	5	VI	freq	52	57	43	4,5
21	-	F	5	VI	freq	46	56	43	16,25
22	-	M	5	VI	freq	47	57	43	10
23	-	F	5	VI	freq	50	62	33	12,6
24	-	M	5	VI	freq	54	56	-	14,16

CERAD: Consortium to Establish a Registry for Alzheimer's Disease; F: female; M: male; NF: neurofibrillary; PMI: post-mortem interval.
